# Dissertation on Dental Caries

**Published:** 1843-09

**Authors:** A. Westcott


### ARTICLE III.

*Dissertation on Dental Caries.* Read before the American Society of Dental Surgeons, at its fourth Annual Meeting; Baltimore, July 20th, 1843. By A. Westcott, M. D., D. D. S.

Few interrogatories are more frequently put to the dental practitioner, than those pertaining to *dental caries*—and few, I am sorry to add, are more frequently, either evaded, or so indefinitely answered. Were the interest upon this subject, among the profession, at all commensurate with its importance, and the general solicitude upon it, correct information would have been more generally diffused, and a greater proportion of the present amount of suffering from its consequences averted.

That erroneous views are entertained, even by members of the profession, upon this point, is fully proven by the fact that, men of unquestionable talents and acquirements, are to be found advocating the most discordant views relative to the pathology of this disease.

While on the one hand we are taught from high authority, that the teeth are, like the soft parts, subject to all the fluctuations of disease and health, in common with the general system, and that dental caries arise chiefly from constitutional causes, we are led by *another*, whose authority is *equally* deserving our respect, to discard almost wholly such causes, and to adopt the belief, that *after the teeth are fully formed*, we are to attribute their decay mainly, if not entirely, to the action of external agents. Theories are in many instances of very little practical utility, or from many of them, no rules are deducible, having a practicable bearing upon the health and comfort of man. For example, it is of little moment, other than a gratification purely mental, whether we explain combustion by supposing that during the process, phlogiston is given off, or oxygen added to the combustible; or again whether we account for calorification on the supposition that it takes place in the lungs, or, at the extremities of the blood-vessels, at the systemic capillary—or, whether we recognize a double or single agency, in explaining electric phenomena, or whether we regard chlorine as a simple or compound substance. In short, many ingenious theories are framed in the various departments of science, which but for the facts and genius elicited, in the necessary investigation, would be an idle waste of time. But while this is true of *many*, most theories pertaining to medicine or surgery, deserve our most careful attention and scrutiny; as on them are frequently based rules of practice of the utmost importance. Indeed, we may as well look for pure waters from a corrupt fountain, as for correct practice in any department of medicine or surgery, where false views are entertained of the origin and nature of the disease. Else why was the Broussain patient suffered to die without a laxative, while it was acknowledged by the most zealous advocates of his theory, that post-mortem examinations exhibited the bowels *distended* with the most acrid and corroding materials? Simply because the Broussain theory admitted of but one "*fous et origo*" of fever, viz. irritation, or inflammation of the mucous membrane of the stomach—hence this viscus must not be *irritated* with a purgative—the alternative being, to leave in contact with, and subject the bowels to the constant impression of, these acrid materials, till either nature or her step-mother death, relieved the patient from his sufferings. If error in *theory* could induce such gross error in *practice*, with so great a man as Broussais, who has long and deservedly been regarded one of the greater lights of his profession, how carefully ought *we* to scan any theory before adopting it, especially where its practical tendencies are deleterious, who can boast, perhaps, of a less elevated genius and profound research, than that possessed by this great and enlightened pathologist.

The same remark relative to theories upon the general practice of surgery and medicine, will apply to *dental* surgery, modified only by the magnitude of the consequences, naturally following the adoption of a *true* or *false* theory.

Let us make an application of this sentiment while we examine some of the grounds taken in support of the opposing theories, to account for dental caries.

Of a subject requiring volumes to do it justice, it is not my purpose, of course, to take an extensive view in a short essay, but shall confine my remarks to the general cause of caries, *after the teeth have received a definite constitution* ; or in other words, to the relative merits of the two opposing theories, the one referring this disease mainly to *constitutional* causes, and the other to the action of *external agents.*

That this disease is far the most destructive of any to which the teeth are subject, will be admitted by every practical dentist, and that the *preventive*, as also to some extent the *curative* treatment, will be directed in accordance with the view taken of the *cause*, is a supposition predicted by common sense, and verified by every day's experience.

How frequently when urging the necessity of greater cleanliness of the mouth and teeth, to our patients, are we met with an "Oh! 'tis of no use to take pains with *my* teeth—my constitution is so bad, or I have taken so much calomel."

Now to reason that when one has a cold, he has a cold in his teeth, or in rheumatism, the teeth partake of the disease, or that they share the morbid impressions made upon the system by the small-pox, or in short every other malady to which *flesh* is heir, is but to contend,

1st. That a diseased system is necessarily attended by a diseased set of teeth—and as it is a generally admitted fact, that they possess no intrinsic restorative powers, it must follow—

2dly. That the teeth of an individual, at any given age, are labouring under the *combined* effects of *all* the diseases with which he has been afflicted up to this period.

Again, if caries depends directly upon disease of the general system, it is equally fair to conclude, that those persons who have inherited good constitutions, and have enjoyed uninterrupted health, must have good teeth.

Before proceeding more minutely to analyze the grounds of this doctrine, I shall lay down a few principles, the *truth* of which is, I believe, generally admitted.

1st. The soft parts of the system, possessing a free circulation, are more subject to attacks of inflammation than any other portion of the body, and also possess the greatest restorative powers—these though frequently attacked with inflammation, are seldom destroyed by gangrene.

2d. The *bones* of the general system, are more *feebly* endowed with circulation, are less subject to inflammatory action, and are less capable of restoring themselves from such attacks.

3d. The bony portion of the teeth is still *more feebly* endowed with vitality, and is proportionably less subject to inflammation, and has *no* intrinsic restorative powers.

4th. The enamel is beyond the direct control of the vital energies, and is of course, *destitute* of the power of self restoration.

If the above principles are correctly stated, and caries to any considerable extent depends upon constitutional causes, it must follow, that while nature has in relation to every other organ of the body, with almost a lavish hand, provided not only against attacks, but also for *restoration*, she has left the *teeth*, so essential in giving symmetry and beauty to the face, and in fitting man to enjoy the luxuries which the kind hand of Providence has spread so bountifully over the surface of the globe,—organs calculated to enhance not only his comfort, but *necessary* for the maintenance of health, organs moreover, when diseased, that subject him to the most excruciating torture, to which his frame is susceptible—*these* she has left to destructive decay, which it is beyond the power of the sufferer to avert.

Nor indeed, are the services of the dentist, much less circumscribed. Though he may offer a *palliative, perhaps* á *remedy,* he cannot, a *cure.*

The hope of affecting a *cure,* without removing the *cause,* which if it be constitutional, is beyond his province, would be as idle, as the attempt, by stopping the *crater,* to extinguish the internal fire of the volcano.

This must have been so regarded by Mr. Koecker and others, who advocate internal causes of caries; for while they have devoted pages to the formation and description of internal caries, they have not given a paragraph relative to any *treatment* calculated to meet the exigency. Were not the reason as above stated so obvious, we should be led to wonder, that while Mr. K. assures us that "it may be laid down as a general rule, that *all* morbific influences, which affect the general health, must *naturally,* more or less affect the teeth, and that constitutional diseases generally, (and especially those of an acute character,) must produce morbid action of some kind *in* the teeth," he does not even hint at any constitutional *treatment* either preventive or curative.

But before bringing any charge against *nature* for withholding her protecting care over organs, in the construction of which she seems to have spared no pains, or adopting a *theory* so mischievous in its practical tendencies; let us see if there are not good reasons for suspecting its truth.

On the supposition then, that dental caries frequently arises from constitutional causes, I would inquire why we would not look for, even *more frequently,* cases of disease in the other bones of the system?

For if caries is ever internal, it must be preceded by inflammation of the bony structure of the tooth, as it cannot be acted upon by external corrosive agents, and as they are by their compactness of structure, less susceptible to inflammatory action, than the bones of the system, of the attacks of *either,* from constitutional causes, those of the *bones* ought to be many fold the most frequent. That cases of inflammation of the bones, often escape observation, is hardly supposable, inasmuch as they are almost uniformly accompanied by intense, deep-seated pains. It is indeed a questionable point, whether ostetis ever terminate in resolution—and without admitting this supposition, the symptoms are always strongly marked. Now the number of cases of diseased bones, compared with the frequency of *dental* caries, would not be a drop of the bucket, whereas if this theory be true, by every known law of the system, they ought by far, to out number them. That it is fair to compare the bones of the system with the teeth, may be inferred from the fact that in organization they are similar, and in composition very nearly the same—the principal difference being the superior compactness of structure of the bones of the teeth, a fact materially favoring the supposition that they would be far less influenced by constitutional causes. But if it be objected that each has a structure and laws peculiar to itself, and that therefore it is unfair to make the one a representative of the other, it may be answered; that the bone of the *teeth themselves* below the crown, which is identical both in composition and organization, are never accused of *internal caries,*—nor indeed of any other, unless the gum naturally protecting it, has been wasted, exposing it to the action of external agents. Now I would ask what good reason can be assigned for *this* portion, being exempt from general disease, while the bony portion of the *crown*, as is contended by Fox, Bell, Koecker and others, partakes of every general morbific agent.

Can we then from the organization of the teeth, or from any known law of the animal economy predict *a priori* that internal caries is of common occurrence. Nay, on the other hand are we not constrained by such considerations to regard it, if it *ever* occurs as exceedingly rare!

From my early reading upon dental surgery, I adopted very naturally the faith of my authors, and became a firm advocate of constitutional caries; nor was it till after several years close attention to the pathology of this disease, that I was led to abandon it altogether.

My present opinion is, that after the teeth are fully formed, internal caries, properly speaking, never occurs; nor can I find any good reason for supposing, *after* this period, that their susceptibility to action of external agents, is materially enhanced.

I have often met with cases of what might perhaps be termed with propriety *deep seated* caries, and when the opening externally was so slight as to require the closest observation to detect it; but never an instance, where the aperture was not sufficient to give ingress to fluids which might act upon the *bone* of the tooth. The enamel being crystalline and of a structure differing from that of the tooth, is very liable either by mechanical violence, or the sudden transitions of temperature, to which it is often exposed to be cracked, so as to be pervious to corroding agents. This effect from the causes alluded to cannot have escaped the observation of any who have been in the habit of carefully examining the teeth.

So common indeed is it for the enamel to be thus checked, that I have seldom examined a set of teeth, with a view to the fact which did not exhibit them in some one or more of the teeth. The cases where the bony structure of the teeth most frequently, suffers from the penetration through these nearly invisible fissures, and which are most liable to be mistaken for internal caries, occur on the lower molars on the outer side directly under the central vertical depression in the tooth.* On sawing in two these teeth horizontally near the centre of the crown, we generally find the enamel covering this depression much thinner than upon the two convex surfaces, upon either side of this point, rendering it more liable to fracture, than upon any other portion of the tooth. This situation it may be observed also, is most favorable to receive the constant effects of any irritating fluid, which may chance to be in the mouth. Caries in this situation is frequently first detected by a dark bluish appearance under the enamel, often requiring the closest observation to see the fissure which gave

* The enamel of the teeth is often so cracked by mechanical violence, and especially on their sides, which come in contact with each other, by the pressure of the organs one against another, as to be penetrated by the secretions of the mouth; but the fissures within the indentations upon their grinding surfaces and in their sides, are in nearly every instance the result of the imperfect formation of this outer covering.—BALT. ED.

rise to it. This description of apparent internal caries, while it applies particularly to the lower molares is by no means confined to them. We find then, no difficulty in satisfactorily accounting for the direct application of those substances to the bony structure of the tooth which are known to act upon it, giving rise to caries.

With this brief notice relative to the liabilities of the bony portion of the teeth to receive the direct action of external agents, we will next examine the effects, upon both the bone and enamel of the teeth of some of the substances which are liable to be brought in contact with them. In order to secure greater accuracy relative to the supposed effects of certain substances, I instituted a series of experiments, conducted in the following manner. I prepared a water bath kept constantly at 98° by a spirit lamp, and regulated by a thermometer. In these were placed vials containing the substances to be tested. In each of these was placed a human tooth, care being taken to select those of as similar organization as possible, and whose enamel was perfect.

This list included about an hundred articles—those most commonly used as food, condiments and medicine. They were carefully examined every forty-eight hours, either by myself or my assistant, Mr. Dalrymple, a young gentleman fully competent to judge of the progress, and the effect carefully noted. The result of only a few of these will be alluded to in this essay, reserving a full report of them for a future article.

If these experiments are calculated to give us a true notion of the action of various substances upon *living* teeth, they furnish much important information, which I have been hitherto unable to obtain. That they do so in respect to their action upon the *enamel*, can hardly be doubted when we consider that this structure is beyond the direct control of the vital powers, and is hence subject to the same effect from chemical action, whether covering dead or living teeth.

And this is far the most important question to settle, as when this natural protection is destroyed, the teeth are always soon ruined.*

* We presume Dr. W. has reference to those cases where its destruction is the result of caries and no means used to prevent the farther progress of the disease, as it is well known to every dental practitioner that teeth often

In respect to the action of these substances upon the living *bone* of the teeth, it must be confessed, there are many modifying circumstances, such as original difference in organization, difference of the age of the subject, and other circumstances connected with vitality, which would vary the action of these chemical agents, yet can it be supposed that the feeble vitality of the tooth is capable to any considerable extent, of *counteracting* the affinities of the substances established by these experiments in respect to dead bone.

But we need not rely solely upon this negative evidence, to believe the bony portion of living teeth *very* susceptible to the action of chemical agents. In many, and perhaps we may say, in most instances of caries in teeth, strictly speaking, there is no cavity till the carious portion be excavated, and what becomes such on being cleaned, was filled with a substance resembling cork, which must be regarded as the animal matter of the tooth, the earthy portion having been taken up by the action of acids, which by the by must be regarded as the main, if not the only substance effecting the teeth by chemical affinity.

Now what is the succession of steps in this destructive process? Do these substances first produce inflammation and death of the bone, and *then* chemical affinity step in to *complete* the process by acting upon its earthy matter? or rather does not the process essentially consist in the acids attack upon the earthy portion; or is not an acid capable of affecting this interstitial decomposition, unaccompanied by inflammation?

If in the cases supposed, the acid is neither the direct agent of inflammation, nor is capable of taking up the earthy matter of the bone without inflammation, why is it that we may predict with certainty, that those acids whose affinity for lime is greater than the acids united with this base in the teeth, will readily destroy them? I have been led to suppose by closely observing the phenomena of caries, that acids not only give rise to inflammation of the bone by their direct attack on, or affinity for its lime, but that this action was not unfrequently unattended by inflammation.

remain healthy and sound through life, after the removal of considerable portions of their enamel, as for example, in those cases where they have been filed for the removal of caries which had attacked their lateral surfaces.

BALT. ED.

The former occurring in highly organized teeth, and the latter result in those teeth whose vital powers were constitutionally feeble. If these observations be correct, it must follow that to a very great extent experiments thus conducted, furnish us with definite information in respect to the chemical action of the substances tested upon living teeth. It is proper to observe however that any given substance by being mixed with the saliva in its different states, and with other substances will have its action modified, though not destroyed, unless it be neutralized by one having for it a chemical affinity. Not only so, but the strength of such affinity must be greater than *either* ingredient of the compound formed for *one* of the constituents of the teeth. For example, suppose tartaric acid in the mouth were met by potash, the action which it would otherwise have upon the teeth might be supposed to be suspended by its affinity for the potash. But even if these two ingredients to unite by simple affinity and as the result tartrate of potash fully formed, yet the effect upon the teeth would be the same in the end, for tartaric acid has a stronger affinity for the lime of the teeth, than for potash, hence by *elective* affinity it would leave its base, and produce its legitimate effect upon the teeth. This accounts for the well known fact that cream of tartar whitens the teeth, and the less known fact that it destroys them.

The idea then, that any substance which of itself is calculated to decompose the teeth, is rendered harmless by being mixed with many others, is not only false, but exceedingly mischievous in its consequences. Believing then that the result of these experiments furnishes us with information which is of practical utility, I shall give a brief summary of them, which may be embodied in the following propositions.

1st. Both vegetable and mineral acids act readily upon the bone and enamel of the teeth.

2d. Alkalies do not act upon the enamel of the teeth; the caustic potash, will readily destroy the bone by uniting with its animal matter.

3d. Salts whose acids have a stronger affinity for the lime of the tooth than for the basis with which they are combined, are decomposed, the acids acting upon the tooth.

4th. Vegetable substances have no effect upon the teeth till after fermentation takes place, but all of them capable of acetic fermentation act readily after this acid is formed.

5th. Animal substances even while in a state of confined putrefaction, act very tardily if at all upon either the bone or enamel. On examining the teeth subjected to such influence, the 20th day of the experiment, no visible phenomena were presented, except a slight deposit upon the surface of a greenish slimy matter, somewhat resembling the green tartar, often found upon teeth in the mouth.

To give a more definite idea of the deleterious agents to which the teeth are exposed, and their consequent liability to be affected by them, we will notice the effect produced by a few of the individual substances, which are more or less liable to be brought in contact with the teeth.

Acetic and cetric acids, so corroded the enamel in forty-eight hours, that much of it was easily removed with the finger nail.

Acetic acid or common vinegar, is not only in common use as a condiment, but is formed in the mouth whenever substances liable to fermentation are suffered to remain about the teeth for any considerable length of time.

Citric acid or lemon juice, though less frequently brought in contact with the teeth, acts upon them still more readily.

Mallic acid or the acid of apples, in its concentrated state also acts promptly upon the teeth.

Muriatic, sulphuric and nitric acids, though largely diluted, soon decompose the teeth—these are in common use as tonics.

Sulphuric and nitric ethers have a similar deleterious effect, as also spirits of nitre—these are common diffusable stimulants in sickness.

The acids of some of the salts also corrode the teeth.

Super tartrate of potash as example, destroyed the enamel very readily—this article is frequently used to form an acidulated beverage.

Raisins so corroded the enamel in twenty-four hours that its surface presented the appearance and was of the consistency of chalk,—why ?

Sugar had no effect till after acetous acid was formed, but then the effect was the same as from this acided when directly applied.

This slight sketch will serve to give some idea of the exposure of the teeth to *external* destructive agents.

I have not included in this catalogue the septic acid, supposed by Dr. Mitchell to be freely generated in certain diseased states of the gums, and which according to his observations is a formidable enemy to the health of the teeth. Now the acid so frequently formed in the stomach in cases of bad digestion, and which is frequently thrown into the mouth in connection with half digested food, in the effort to relieve the stomach from painful tension.

Judging from the effects of this latter acid upon the throat and mouth, it is safe to conclude that it would act destructively upon the teeth. It is this, I have no doubt, which lays the foundation for that rapid destruction of the teeth which is so often observed in pregnancy, but which Mr. Koecker ascribes to a peculiar irritable state of the system.

I shall conclude this list of teeth destroyers by a brief allusion to the acid state of the saliva in certain diseases as given by M. Donna, of Paris, and reviewed by Dr. Johnson, in the Medico Chirurgical Review, vol. 1, for 1836.

Endorsed as this authority is by Dr. Johnson, it has the highest claims to our respect.

M. Donna assumes that the saliva in its normal state is uniformly alkaline.

This conclusion as shown by his quotations, is corroborated by several other distinguished physiologists.

The pathological condition of the stomach indicated by an acid state of the saliva, is irritation or inflammation of its mucous membrane, and as he contends, this condition of the stomach uniformly induces, or is accompanied by acidity of the saliva. Besides giving the result of his laborious experiments, in arriving at these conclusions he has narrated a large number of cases illustrative of the corresponding change from acidity to alkalinity as the patient recovered from the disease. In many cases he describes the effect of the saliva upon letmus paper as prompt as would be made by strong vinegar. If these observations are correct, while they shew us the beautiful provisions of nature, in making the normal state of the saliva alkaline, thus constituting it in respect to the teeth, not only harmless, but an efficient protection against the acids so liable to be brought in contact with or generated about them, it points most clearly to the treatment best calculated to counteract their effects.

From this brief allusion to some of the external destructive agents which may singly or in combination act upon the teeth, we are led to wonder, not that we have so *much* disease from so *few* causes, but that we have so *little* from so *many* causes.

